# Radiomic Characterization of Adrenal Incidentalomas on NECT: Retrospective Exploratory Study and Systematic Review

**DOI:** 10.3390/jimaging12040151

**Published:** 2026-03-30

**Authors:** Pasquale Frisina, Paolo Ricci, Filippo Valentini, Daniela Messineo

**Affiliations:** 1 Department of Radiological, Oncological and Pathological Sciences, Sapienza University, 00161 Rome, Italy; 2 Department of Sense Organs, Sapienza University, 00161 Rome, Italy

**Keywords:** radiomics, feature extraction, adrenal incidentaloma, pheochromocytoma

## Abstract

Radiomics may aid the noninvasive characterization of adrenal incidentalomas; however, reproducibility is limited by methodological heterogeneity. In this retrospective, single-center, exploratory study, we tested whether radiomic features from baseline non-enhanced computed tomography (NECT) discriminate benign from malignant/metastatic adrenal lesions and contextualized results with a PRISMA 2020 systematic review (PubMed/Scopus 2017–2025; PROSPERO CRD420251276627). Thirty-three patients (36 lesions: 12 lipid-rich adenomas, 9 lipid-poor adenomas, 6 pheochromocytomas, 7 malignant/metastatic lesions, 2 myelolipomas) were included; myelolipomas were excluded from primary comparisons. Two abdominal radiologists performed consensus 3D segmentation on NECT. Using LIFEx (v7.8.0) and IBSI definitions, 42 features were extracted and z-score standardized. LASSO selected four heterogeneity descriptors: First-order Entropy, gray-level co-occurrence matrix (GLCM) entropy, gray-level size zone matrix (GLSZM) non-uniformity, and neighboring gray tone difference matrix (NGTDM) busyness. Heterogeneity increased from lipid-rich adenomas to pheochromocytomas and malignant/metastatic lesions (Kruskal–Wallis, all *p* < 0.001. Pairwise separability, measured using the Vargha–Delaney A index (VDA) as a rank-based measure of separability, was highest for lipid-rich adenomas versus malignant/metastatic lesions (0.93), intermediate for lipid-poor adenomas versus pheochromocytomas (0.73), and lowest for lipid-rich versus lipid-poor adenomas (0.64). The review identified 18 eligible CT radiomics studies that consistently reported higher entropy/non-uniformity in pheochromocytomas and malignant lesions than in lipid-rich adenomas. Global heterogeneity metrics on NECT may complement conventional CT criteria in indeterminate lesions; external validation with robust reference standards is needed in larger, multicenter cohorts with harmonization.

## 1. Introduction

Adrenal incidentalomas are common imaging findings and are benign in most cases; however, a subset requires further diagnostic assessment to exclude hormonally active lesions or malignancy [1,2,3]. Non-contrast CT attenuation (Hounsfield units, HU) and contrast-enhanced washout studies remain key diagnostic tools, yet a proportion of lesions remain indeterminate in routine practice [1,2]. Radiomics is a quantitative imaging approach that extracts high-dimensional descriptors of lesion heterogeneity and texture beyond visual assessment [4]. Nevertheless, published evidence in adrenal imaging radiomics is often limited by small cohorts and methodological heterogeneity (acquisition, segmentation, discretization, feature extraction, and analysis), which can hinder reproducibility and generalizability [5,6]. In this context, standardized radiomics workflows and transparent reporting, including IBSI-aligned feature definitions and software, are needed to improve comparability across studies [7].

Therefore, we performed a single-center, retrospective, exploratory radiomics analysis of baseline NECT and complemented the original data with a PRISMA-based systematic review (PROSPERO: CRD420251276627) to contextualize feature-level trends reported in the literature [8,9]. Our objective was not to propose a validated diagnostic classifier, but to test whether global heterogeneity metrics on NECT show consistent, interpretable gradients across common adrenal lesion categories, thereby providing a hypothesis-generating framework for future external validation.

## 2. Materials and Methods

### 2.1. Study Design and Population

This was a single-center retrospective exploratory study. The database was queried between October 2024 and May 2025 to retrieve patients with available abdominal CT examinations, including scans acquired before this query window. The reference standard was defined using a composite approach including histopathology when available, biochemical evaluation when indicated, and imaging follow-up. No missing data were present for the analyzed variables. All benign lesions without histopathological confirmation showed radiological stability on imaging follow-up of at least 12 months. Baseline non-enhanced CT (NECT) examinations acquired at the time of initial lesion detection and prior to any treatment were used for radiomic analysis.

Patients were eligible if they had an adrenal incidentaloma detected on CT and sufficient imaging quality for segmentation and feature extraction. Lesions were classified as lipid-rich adenoma, lipid-poor adenoma, pheochromocytoma, or malignant/metastatic lesion based on a composite reference standard that integrated imaging features, biochemical assessment when indicated, and longitudinal follow-up. Myelolipomas were recorded but excluded from primary group comparisons due to their characteristic fat-containing appearance and the limited number of cases.

### 2.2. Radiomic Feature Extraction

Only NECT images were analyzed. All NECT examinations were performed on a single multidetector CT scanner (120 kVp, automatic tube current modulation, collimation 0.6–1.0 mm, reconstructed slice thickness 2–3 mm) using a soft-tissue kernel. Images were exported in DICOM format for radiomic processing.

Lesions were manually segmented in three dimensions (3D) on axial NECT images by two abdominal radiologists (with 8 and 15 years of experience in abdominal imaging) working in consensus. Both readers jointly reviewed each case and generated a single consensus segmentation for each lesion. Particular care was taken to exclude surrounding fat, vessels, and adjacent organs to minimize partial-volume effects and avoid contamination of radiomic features. Radiomic feature extraction was performed using LIFEx software (v7.8.0) [10] according to IBSI feature definitions [7]. A total of 42 features were initially extracted, including first-order intensity features, gray-level co-occurrence matrix (GLCM), gray-level size zone matrix (GLSZM), and neighboring gray tone difference matrix (NGTDM) features.

**Figure A1:**
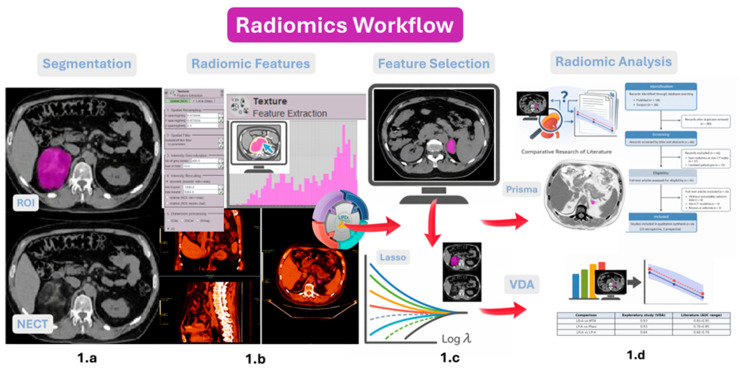
Schematic representation of the study design, including ROI segmentation on NECT images (**1a**), radiomic feature extraction (**1b**), LASSO-based feature selection (**1c**), systematic review conducted according to PRISMA guidelines, and subsequent statistical analysis (**1d**). Arrows indicate workflow progression, different colors represent distinct processing steps, and the symbol ‘?’ indicates decision or evaluation points. NECT: non-enhanced computed tomography; ROI: region of interest.

Image intensities were discretized using a fixed-bin approach with 64 bins as implemented in LIFEx for CT imaging, applied to HU values within each segmented lesion. No additional intensity clipping, voxel resampling, or spatial interpolation was applied because all examinations were acquired on a single scanner with a consistent acquisition and reconstruction protocol. After feature extraction, all radiomic features were standardized using z-score transformation and rescaled to the 0–1 range to support robust statistical comparisons across lesion groups. The overall study workflow is summarized in Figure A1 (Appendix B).

### 2.3. Feature Selection and Statistical Analysis

To reduce feature redundancy and multicollinearity in this exploratory setting, feature selection was performed using the Least Absolute Shrinkage and Selection Operator (LASSO) regression [11]. LASSO was applied in a supervised manner using a binary outcome (benign versus malignant/metastatic lesions) solely to guide feature selection, not to develop or validate a predictive classifier. All preprocessing steps, including feature standardization and LASSO-based feature selection, were performed on the full dataset, as no data splitting or predictive modeling was intended in this exploratory analysis. The regularization parameter (lambda) was selected using 5-fold cross-validation to minimize the cross-validated error. The four selected features were then compared across lesion categories using non parametric tests. The selected radiomic features are summarized in Table 1. Radiomic feature extraction and statistical analysis were performed using dedicated software and Python libraries, as detailed in Appendix A.

Overall group differences were assessed using the Kruskal–Wallis test. Pre-specified pairwise comparisons were performed using Dunn’s test with Bonferroni correction to control for multiple comparisons. Effect sizes were summarized using Cliff’s delta and the Vargha–Delaney A index (VDA), a probabilistic measure of separability related to Cliff’s delta [12]. The Vargha–Delaney A index (VDA) is a nonparametric, rank-based measure of stochastic superiority that quantifies the probability that a randomly selected observation from one group has a higher value than a randomly selected observation from another group.

### 2.4. Segmentation Robustness and Reproducibility

All segmentations were performed in consensus by two abdominal radiologists using a standardized 3D delineation protocol. However, no independent repeated segmentations were performed, and therefore interobserver reproducibility could not be quantified using intraclass correlation coefficients (ICC). Given that radiomic features are known to be sensitive to segmentation variability, the lack of reproducibility assessment represents a methodological limitation.

### 2.5. Systematic Review (Protocol Registration and Search Strategy)

**Figure A2:**
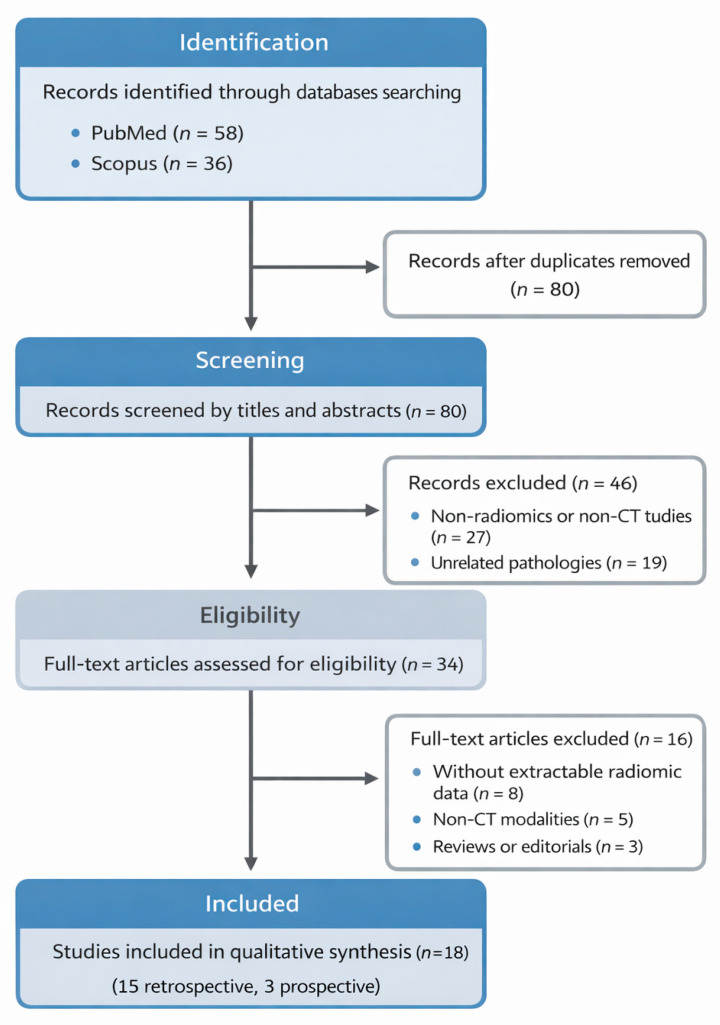
PRISMA 2020 flow diagram of study selection [8].

The systematic review protocol was registered in PROSPERO (CRD420251276627) [9]. PubMed and Scopus were searched for English-language articles published from 1 January 2017 to 31 May 2025. Reference list checking (backward citation searching) and forward citation searching were also performed. Titles/abstracts and full texts were screened independently by two reviewers, with disagreements resolved by discussion and consensus. As pre-specified in the protocol, the study risk of bias was not assessed. The review is reported in accordance with PRISMA 2020 [8]. The study selection process is illustrated in Figure A2 (Appendix B).

The search identified 94 records (58 from PubMed, 36 from Scopus). After removing 14 duplicates, 80 unique records were screened. Title/abstract screening excluded 46 records; 34 full texts were assessed for eligibility, and 18 studies met the inclusion criteria and were included in the qualitative synthesis.

Inclusion criteria were radiomic analysis on NECT images of adrenal lesions with a clinical or pathological reference standard and extractable feature-level data; when multiphasic CT was used, studies were included only if NECT-derived radiomic data were reported separately. Exclusion criteria comprised non-CT imaging, non-NECT feature extraction, case reports, narrative reviews, and studies without extractable radiomic feature data. Included studies enrolled between 25 and 310 patients and predominantly used manual 3D segmentation, IBSI-aligned software platforms (e.g., LIFEx or PyRadiomics), and feature selection methods such as LASSO.

## 3. Results

The final cohort included 33 patients with 36 adrenal lesions; three patients presented with bilateral involvement. The mean age was 58.4 years (range 41–77), with 18 women (54.5%) and 15 men (45.5%). Lesion composition included 12 lipid-rich adenomas (33.3%), 9 lipid-poor adenomas (25.0%), 6 pheochromocytomas (16.7%), 7 malignant or metastatic lesions (19.4%), and 2 myelolipomas (5.6%). As specified in advance, myelolipomas were excluded from primary group comparisons. Radiomic features showed a progressive increase in heterogeneity from lipid-rich adenomas to pheochromocytomas and malignant/metastatic lesions. The distribution of radiomic feature values across lesion groups is reported in Table 2.

### Statistical Analysis

**Figure A3:**
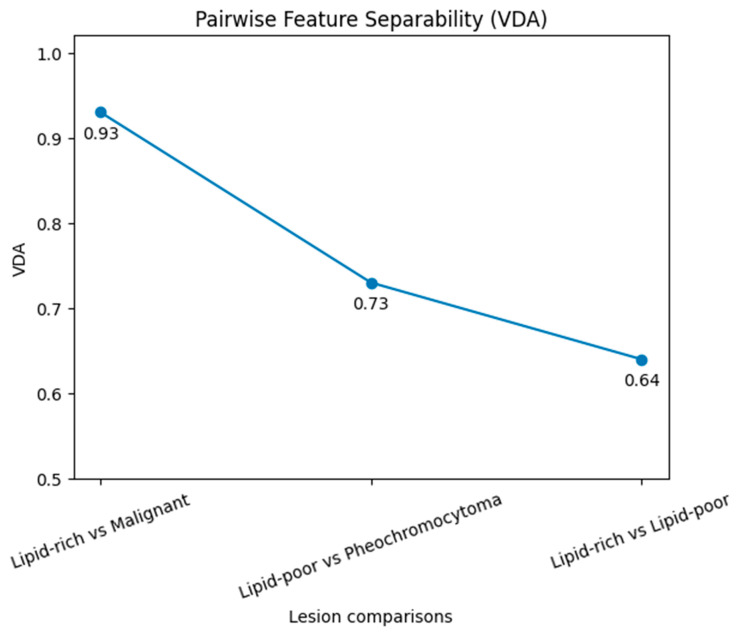
Pairwise feature-level separability across lesion groups measured using the Vargha–Delaney A index (VDA). The plot illustrates a progressive gradient of separability, with higher values indicating greater distributional differences between groups.

Statistical analysis confirmed significant differences across adrenal lesion groups for all radiomic features examined. Pairwise comparisons and effect sizes are summarized in Table 3. The Kruskal–Wallis test indicated global significance for First-order Entropy, GLCM Entropy, GLSZM Non-uniformity, and NGTDM Busyness (all *p* < 0.001). Post hoc pairwise comparisons using Dunn’s test with Bonferroni correction demonstrated a progressive gradient of differentiation. The most significant differences were observed between lipid-rich adenomas and malignant lesions (all *p* < 0.001), with very large effect sizes (Cliff’s delta = 0.80–0.88). Comparisons between lipid-poor adenomas and pheochromocytomas yielded moderate differences (*p* = 0.03–0.05, Cliff’s delta 0.41–0.47). By contrast, lipid-rich versus lipid-poor adenomas did not reach conventional statistical significance (*p* = 0.06–0.09), with small effect sizes (Cliff’s delta 0.25–0.29) (Table 3). To provide a probabilistic measure of discrimination, the Vargha–Delaney A index (VDA) was calculated for the main pairwise contrasts. Pairwise separability results are reported in Table 4. The VDA values are presented as preliminary, rank-based estimates of feature separability and should not be interpreted as measures of diagnostic performance. As no predictive model was developed, conventional performance metrics such as AUC, sensitivity, and specificity were not calculated. VDA values reached 0.93 for lipid-rich adenomas versus malignant lesions, 0.73 for lipid-poor adenomas versus pheochromocytomas, and 0.64 for lipid-rich versus lipid-poor adenomas. These values indicate strong, moderate, and weak feature-level separability, respectively. In particular, separability was highest for lipid-rich adenomas versus malignant lesions (VDA = 0.93), intermediate for lipid-poor adenomas versus pheochromocytomas (VDA = 0.73), and lowest between lipid-rich and lipid-poor adenomas (VDA = 0.64), indicating a progressive gradient across lesion types. The pairwise feature-level separability across lesion groups is visually illustrated in Figure A3. (Appendix B). The integrated literature review, conducted according to PRISMA-inspired criteria, identified 94 studies, of which 18 met the inclusion criteria (2017–2025). Fifteen studies were retrospective, and three were prospective, with sample sizes ranging from 25 to 310 patients. LIFEx, PyRadiomics, and proprietary software were the most frequently employed platforms.

VDA values indicated strong (0.93), moderate (0.73), and weak (0.64) feature-level separability across the three central lesion contrasts. The most frequently analyzed features were GLCM, GLSZM, NGTDM, and first-order intensity metrics. Across studies, pheochromocytomas and malignant lesions consistently showed higher entropy and non-uniformity than lipid-rich adenomas. Table 5 summarizes the comparison between the present exploratory study and the published literature, highlighting the convergence of findings across independent cohorts. Overall, these findings indicate that heterogeneity-related radiomic descriptors exhibit consistent behavior across adrenal lesion subtypes, reinforcing their potential as complementary markers for the non-invasive characterization of indeterminate incidentalomas. VDA was used exclusively as an exploratory, rank-based metric to quantify pairwise feature separability and was not intended to assess diagnostic or predictive performance, which would require validated classification models.

## 4. Discussion

This exploratory, single-center study integrated original NECT radiomics data with a PRISMA-based systematic review (PROSPERO: CRD420251276627) [8,9]. We observed a consistent heterogeneity gradient across adrenal lesion categories: lipid-rich adenomas showed the lowest entropy and non-uniformity values, lipid-poor adenomas showed intermediate values, and pheochromocytomas and malignant/metastatic lesions showed the highest heterogeneity. This pattern is biologically plausible and aligns with prior CT radiomics studies and meta-analytic evidence in adrenal lesion characterization [6]. The individual studies and related literature considered in the systematic review, along with the contextual comparisons, are listed in References [13,14,15,16,17,18,19,20,21,22,23,24,25,26,27,28,29,30,31,32,33,34,35,36,37,38,39,40,41,42,43,44].

Methodologically, we employed 3D manual consensus segmentation and an IBSI-aligned feature-extraction workflow using LIFEx [7,10]. By focusing on global (whole-lesion) heterogeneity metrics, the analysis reduces sensitivity to local HU fluctuations and minor boundary differences that can affect voxel-wise or map-based approaches on NECT. The single-scanner acquisition and consistent reconstruction protocol further limited technical variability, supporting consistency with feature-level trends reported in the literature (Table 6). These comparisons are descriptive and intended to provide qualitative context only; they do not imply methodological equivalence with performance metrics such as AUC derived from predictive models. Literature ranges are approximate and derived from heterogeneous studies, and are therefore not directly comparable due to differences in acquisition, preprocessing, and feature extraction pipelines… Compared with existing imaging approaches, this study does not aim to demonstrate superior diagnostic performance, but rather to confirm consistent feature-level behavior across lesion types. The potential incremental value lies in supporting the interpretation of indeterminate lesions rather than replacing established diagnostic criteria. Clinically, radiomics offers limited added value for lesions with unequivocal imaging appearance (e.g., lipid-rich adenomas and myelolipomas), but may complement conventional CT criteria in indeterminate lesions where diagnostic overlap persists despite guideline-based assessment [1,2]. In clinical practice, these findings suggest that heterogeneity-related radiomic features may support the characterization of indeterminate adrenal lesions, particularly when conventional CT criteria are inconclusive. However, no specific decision thresholds can be proposed at this stage due to the exploratory design. Importantly, this work is feature-level and hypothesis-generating: we did not develop or validate a classifier, and external validation integrating biochemical assessment and standardized reporting is required before clinical implementation.

This study has some limitations. The sample size was modest, reflecting its exploratory design, and larger prospective cohorts are needed to consolidate these findings. No external validation cohort was available, and the findings should therefore be considered preliminary and not directly generalizable beyond this exploratory setting. In addition, the relatively small sample size combined with the high dimensionality of radiomic features introduces a substantial risk of overfitting. In such settings, feature selection methods, including LASSO, may be unstable, leading to variability in the selected features depending on the specific sample composition. Moreover, no feature selection stability analysis (e.g., resampling or leave-one-out procedures) was performed. Therefore, the robustness of the selected feature set cannot be assessed, and the findings should be considered exploratory and hypothesis-generating.

The relatively small sample size reflects the exploratory nature of the study and limits statistical power and generalizability. Therefore, the results should be interpreted as hypothesis-generating and require confirmation in larger multicenter cohorts with external validation. Lesion segmentation was performed manually, which may introduce inter-observer variability; however, a standardized protocol and joint review by two experienced radiologists were employed to minimize this effect. Additionally, the use of non-enhanced CT (NECT) limits the characterization of lipid content, and a monocentric retrospective design may reduce the generalizability of the results. Formal reproducibility metrics, such as the intraclass correlation coefficient (ICC), were not assessed. Finally, although a consistent reconstruction protocol was used, variability in reconstruction algorithms could influence radiomic feature stability in multicenter settings. Given the limited sample size, LASSO-based feature selection was used for exploratory dimensionality reduction rather than for predictive modeling; the results should be interpreted accordingly. The Vargha–Delaney A index (VDA) [12] was used as a preliminary, rank-based measure of group discrimination. However, it should not be interpreted as equivalent to AUC in multi-class comparisons, and its results are intended only as an exploratory indication of feature separability. Because no predictive or multivariable classification model was developed, conventional diagnostic metrics, such as AUC, were not directly evaluated. A formal risk-of-bias assessment was not performed, as pre-specified in the PROSPERO protocol. This work aimed primarily to provide an exploratory, feature-level synthesis of radiomic heterogeneity patterns rather than to estimate diagnostic accuracy or develop predictive models. To enhance transparency and reproducibility, we reported the radiomics workflow using IBSI-aligned terminology and LIFEx (v7.8.0) [7]. Future studies that develop and validate prediction models should follow established reporting guidelines and checklists (e.g., CLAIM and TRIPOD-AI) and include external validation and reproducibility analyses (e.g., ICC-based feature stability) [45,46].

## 5. Conclusions

NECT radiomics identified a reproducible heterogeneity gradient across major adrenal incidentaloma categories, with increasing entropy and non-uniformity from lipid-rich adenomas to pheochromocytomas and malignant/metastatic lesions. In this exploratory cohort, feature distributions and separability measures showed qualitative agreement with trends reported in the literature. Radiomics appears most relevant for indeterminate lesions (e.g., lipid-poor adenomas versus pheochromocytomas or metastases) where conventional CT criteria may be insufficient. Larger multicenter studies with robust assessments of reproducibility and external validation are warranted.

## Figures and Tables

**Table 1:** Radiomic features selected by LASSO and used for group comparisons.

Feature Name	Category	Metric/Description	Notes
First-order Entropy	First-order	Measures the heterogeneity of intensity values within the VOI	Higher values indicate greater disorder among voxels
GLCM Entropy	Texture-GLCM	Entropy calculated from the Gray Level Co-occurrence Matrix	Reflects the spatial complexity of intensity patterns
GLSZM non-uniformity	Texture-GLSZM	Non-uniformity of gray level zones	Higher non-uniformity indicates greater heterogeneity
NGTDM Busyness	Texture-NGTDM	“Busyness” Neighbourhood Gray Tone Difference Matrix	Indicates how rapidly gray levels change along runs

**Table 2:** Median values with interquartile ranges (25th–75th percentiles) of the four selected radiomic features by lesion group (normalized to 0–1).

Feature	LR-A	LP-A	Pheo	MTH
First-order	0.22	0.46	0.63	0.71
Entropy	[0.18–0.27]	[0.41–0.52]	[0.59–0.68]	[0.66–0.75]
GLCM	0.25	0.44	0.61	0.72
Entropy	[0.20–0.30]	[0.39–0.49]	[0.56–0.65]	[0.67–0.76]
GLSZM	0.28	0.52	0.74	0.81
Non-uniformity	[0.24–0.32]	[0.47–0.58]	[0.70–0.79]	[0.77–0.85]
NGTDM	0.20	0.41	0.59	0.65
Busyness	[0.17–0.23]	[0.37–0.46]	[0.54–0.63]	[0.61–0.70]

**Table 3:** Pairwise comparisons (Dunn’s test) and effect sizes (Cliff’s delta) for the four selected radiomic features.

Feature	Kruskal–Wallis *p*-Value	Pairwise Comparison	*p*-	Cliff’s Delta	Effect Size
First-order Entropy	<0.001	Lipid-rich vs. Malignant	<0.001	0.85	Very large
First-order Entropy		Lipid-poor vs. Pheochromocytoma	0.04	0.45	Moderate
First-order Entropy		Lipid-rich vs. Lipid-poor	0.07	0.28	Small
GLCM Entropy	<0.001	Lipid-rich vs. Malignant	<0.001	0.82	Very large
GLCM Entropy		Lipid-poor vs. Pheochromocytoma	0.05	0.41	Moderate
GLCM Entropy		Lipid-rich vs. Lipid-poor	0.09	0.26	Small
GLSZM non-uniformity	<0.001	Lipid-rich vs. Malignant	<0.001	0.88	Very large
GLSZM non-uniformity		Lipid-poor vs. Pheochromocytoma	0.03	0.47	Moderate
GLSZM non-uniformity		Lipid-rich vs. Lipid-poor	0.08	0.29	Small
NGTDM Busyness	<0.001	Lipid-rich vs. Malignant	<0.001	0.80	Very large
NGTDM Busyness		Lipid-poor vs. Pheochromocytoma	0.04	0.43	Moderate
NGTDM Busyness		Lipid-rich vs. Lipid-poor	0.06	0.25	Small

**Table 4:** Pairwise separability between lesion groups measured using the Vargha–Delaney A index (VDA).

Comparison	VDA	Interpretation
LR-A vs. MTH	0.93	Strong separability
LP-A vs. Pheo	0.73	Moderate separability
LR-A vs. LP-A	0.64	Weak separability

**Table 5:** Studies included in the systematic review (2017–2025) and key methodological features.

Author (Year)	Study Design	Population	CT Protocol	Analytical Approach	Main Features	Main Outcome
[13]	Retrospective	Pheo vs. lipid-poor adenoma	Unenhanced CT	Texture analysis	First-order, GLCM	Texture metrics differentiated lesion types
[14]	Retrospective	Subclinical pheo vs. adenoma	Unenhanced CT	ML-based texture	First-order, GLCM, GLSZM	ML-radiomics improved discrimination
[15]	Retrospective	Benign vs. metastatic	Unenhanced CT	Texture analysis	GLCM, GLSZM	Texture-separated metastases vs. benign
[16]	Retrospective	Benign vs. malignant	Unenhanced CT	Radiomics	GLCM, GLSZM	Malignant higher heterogeneity
[17]	Retrospective	Small indeterminate tumors	Adrenal protocol CT	ML-radiomics	First-order, NGTDM	Radiomics improved classification
[18]	Retrospective	Adenoma vs. carcinoma	CT	Radiomics	First-order, GLCM	Entropy distinguished groups
[19]	Retrospective	Lipid-poor adenoma vs. non-adenoma	Unenhanced CT	Radiomics	First-order, GLSZM	Added value beyond attenuation
[20]	Retrospective	Pheo vs. adenoma	Unenhanced CT	ML-radiomics	First-order, GLCM	ML enhanced diagnosis
[21]	Retrospective	Cortisol-secreting vs. non-secreting adenomas	CT	ML-texture	Texture features	Radiomics reflected function
[22]	Retrospective	Metastases vs. adenoma	Portal venous CT	Radiomics	GLCM, GLSZM	Improved metastasis identification
[23]	Retrospective	Adenoma vs. metastasis	Dual-energy CT	Radiomics	GLSZM, NGTDM	Radiomics diagnostic accuracy
[24]	Retrospective	Mixed adrenal lesions	CT	ML-texture	Texture metrics	ML improved accuracy
[25]	Pro-spective	L-PA vs. Pheo	Multiphase CT	ML-radiomics	First-order, GLCM, GLSZM	Radiomics features distinguished high accuracy
[26]	Retrospective	Lipid-poor adenomas	Unenhanced CT	Radiomics	First-order, GLCM, GLSZM	Radiomics > density metrics
[27]	Prospective	Adrenal lesions	Unenhanced CT	Cross-software radiomics	GLCM, GLSZM	Stable across platforms
[28]	Prospective	Automatic AI detection	CT	Deep learning	CNN features	High accuracy for detection
[29]	Retrospective	Lung CA + incidentalomas	Unenhanced CT	Radiomic nomogram	GLSZM, GLCM	Predicted metastases
[30]	Retrospective	Pheo vs adenomas	Unenhanced CT	Texture analysis	First-order, GLCM	Radiomics differentiated adenoma/pheo

**Table 6:** Qualitative comparison of feature-level trends with previously published radiomics studies (2017–2025).

Feature	Lesion Group	Exploratory Study	Median [IQR]	Literature	Consistency
First-order Entropy	Lipid-rich adenomas	0.22	0.18–0.27	0.40–0.55	Consistent (low entropy)
First-order Entropy	Lipid-poor adenomas	0.46	0.41–0.52	0.55–0.70	Consistent (intermediate values)
First-order Entropy	Pheochromocytomas	0.63	[0.59–0.68]	0.65–0.80	Consistent (high entropy)
First-order Entropy	Malignant/metastatic	0.72	[0.66–0.75]	0.72–0.82	Consistent (maximum heterogeneity)
GLCM Entropy	Lipid-rich adenomas	0.25	0.20–0.30	0.40–0.55	Consistent
GLCM Entropy	Lipid-poor adenomas	0.49	[0.39–0.49]	0.55–0.70	Consistent
GLCM Entropy	Pheochromocytomas	0.61	[0.56–0.65]	0.65–0.78	Consistent
GLSZM non-uniformity	Lipid-rich adenomas	0.28	0.24–0.32	0.25–0.35	Consistent
GLSZM non-uniformity	Lipid-poor adenomas	0.52	0.47–0.58	0.50–0.60	Consistent
GLSZM non-uniformity	Pheochromocytomas	0.74	0.70–0.79	0.65–0.80	Consistent
GLSZM non-uniformity	Malignant/metastatic	0.81	0.77–0.85	0.70–0.85	Consistent
NGTDM Busyness	Lipid-rich adenomas	0.20	0.17–0.23	0.18–0.28	Consistent
NGTDM Busyness	Lipid-poor adenomas	0.41	0.37–0.46	0.38–0.50	Consistent
NGTDM Busyness	Pheochromocytomas	0.59	0.54–0.63	0.55–0.65	Consistent
NGTDM Busyness	Malignant/metastatic	0.65	0.61–0.70	0.60–0.70	Consistent

## Data Availability

The data presented in this study are available from the corresponding author upon reasonable request. The data are not publicly available due to privacy and institutional restrictions.

## Abbreviations

The following abbreviations are used in this manuscript:

PROSPERO	International prospective register of systematic reviews
PRISMA	Preferred Reporting Items for Systematic Reviews and Meta-Analyses
MTH	malignant/metastatic lesion
Pheo	pheochromocytoma
LP-A	lipid-poor adenoma
LR-A	lipid-rich adenoma
ICC	intraclass correlation coefficient
LASSO	least absolute shrinkage and selection operator
VDA	Vargha–Delaney A index
ROI	region of interest
NECT	non-enhanced computed tomography
NGTDM	neighboring gray tone difference matrix
GLSZM	gray-level size zone matrix
GLCM	gray-level co-occurrence matrix
IBSI	Image Biomarker Standardization Initiative
HU	Hounsfield unit
CT	computed tomography
AUC	area under the curve

## Appendix A. Appendix Libraries Used for Statistical and Radiomic Analysis in Python 3.10

NumPy (v1.24.3)
Pandas (v2.0.3) SciPy (v1.11.4)
Scikit-learn (v1.3.0)
Matplotlib (v3.7.2) & Seaborn (v0.12.2)
